# Supplementary material for: Fasting, food and farming: Value chains and food taboos in Ethiopia
Source: PLoS One. 2021 Dec 9;16(12):e0259982. doi: 10.1371/journal.pone.0259982 (PMC8659323; doi:10.1371/journal.pone.0259982)
Supplement: S1 Appendix — (DOCX) [file pone.0259982.s001.docx]

| **Variable** | **Unit** | **Household** | **Youngest child** |
| --- | --- | --- | --- |
| **Household controls** |  |  |  |
| Monthly total income | ‘000 ETB/month | 3.68 (4.52) |  |
| Household composition: |  |  |  |
| *Infants (<1 year old)* | # | 0.08 (0.28) |  |
| *Children (1-6 years old)* | # | 0.63 (0.79) |  |
| *Children (7-9 years old)* | # | 0.52 (0.66) |  |
| *Adolescents (10-19 years old)* | # | 2.11 (1.52) |  |
| *Adults (20-64 years old)* | # | 2.44 (1.12) |  |
| *Elderly (65+ years old)* | # | 0.24 (0.51) |  |
| Sex household head: male | 0/1 | 0.92 |  |
| Age household head | years | 48.95 (13.71) |  |
| Schooling household head | years | 3.76 (4.27) |  |
| Marital status household head: |  |  |  |
| *Married* | 0/1 | 0.91 |  |
| *Widowed* | 0/1 | 0.06 |  |
| *Divorced/separated* | 0/1 | 0.02 |  |
| *Single* | 0/1 | 0.01 |  |
| Religious affiliation household head: |  |  |  |
| *Orthodox Christian* | 0/1 | 0.85 |  |
| *Protestant* | 0/1 | 0.12 |  |
| *Catholic* | 0/1 | 0.01 |  |
| *Muslim* | 0/1 | 0.02 |  |
| *Traditional faith* | 0/1 | 0.00 |  |
| *Other religion* | 0/1 | 0.00 |  |
| Ethnicity household head: |  |  |  |
| *Oromo* | 0/1 | 0.86 |  |
| *Amhara* | 0/1 | 0.12 |  |
| *Tigrayan* | 0/1 | 0.00 |  |
| *Gurage* | 0/1 | 0.01 |  |
| *Other* | 0/1 | 0.01 |  |
| Total dairy production | l/month | 400 (597) |  |
| Total cows | # | 2.82 (2.17) |  |
| Cross-bred cows | # | 1.14 (2.10) |  |
| Cooperative member | 0/1 | 0.04 |  |
| Access to Addis market | 0/1 | 0.35 |  |
| Remoteness | hours | 1.98 (1.46) |  |
| Orthodox concentration (village) | % | 84.45 (20.64) |  |
| **Youngest child controls** |  |  |  |
| Age | years |  | 2.01 (1.32) |
| Sex: male | 0/1 |  | 0.56 |
| Breastfed yesterday | 0/1 |  | 0.56 |
| Vitamins yesterday | 0/1 |  | 0.04 |
| ORS (hydration) yesterday | 0/1 |  | 0.03 |
| Observations |  | 870 | 317 |

*Note.* When applicable, means are shown with standard deviations in parentheses.
