# Supplementary material for: Fasting, food and farming: Value chains and food taboos in Ethiopia
Source: PLoS One. 2021 Dec 9;16(12):e0259982. doi: 10.1371/journal.pone.0259982 (PMC8659323; doi:10.1371/journal.pone.0259982)
Supplement: S2 Appendix — (DOCX) [file pone.0259982.s002.docx]

Food production and consumption patterns vary with agricultural seasons in many countries. In Ethiopia, religion, in addition to weather cycles, also affects the dairy sector. Whereas the annual rainfall pattern affects dairy supply due to its impact on feed availability, the periodic occurrence of Orthodox fasting and feasts causes variation in demand for milk and other dairy products. We match both types of seasonality in Figure B1 and show how both sources of variation in production and demand over a period of 12 months preceding the survey impact dairy cow productivity (Fig B2); the number of cows in milk, both local and cross-bred (Fig B3); and average market prices for different types of dairy products, as reported by survey participants (see Fig 2 in the main text).

Figure B1 outlines the rainfall pattern and associated agricultural seasons as well as the timing of the main Orthodox fasts and feasts throughout the year. Seasonal rainfall distribution varies across different areas in Ethiopia, with some areas having spring rains. But generally most of the precipitation falls between June and September. The dry period usually takes places from February through May. Livestock feed availability follows this rainfall pattern, with feed being abundantly available from September to December, after which availability decreases. Critical feed shortages may occur between April and June [1].

This pattern of feed availability matches the seasonal pattern in dairy cow productivity in our sample (Fig B2). Daily milk production per cow is at its lowest average level between January and June (2.6 liters/cow), after which it slowly increases to about 3.3 liters per cow for the period between September and December. Milk producing households seem to align their production practices with the agricultural seasons. As feed availability increases between June and December, farmers hold a rising percentage of cows in milk (Fig B3).

Alternatively, one could argue that producers align their milk production to periods of reduced demand caused by fasting periods. They can do this by making sure that the dry periods of their cows coincide with long Orthodox fasting events. Some Orthodox fasting events overlap with periods of potential feed shortage problems (Fig B1). This is most obvious for the Apostles fast, which overlaps with the lean season during which the probability of acute livestock feed shortages is high. But also during the Lent fasting period, feed availability continuously decreases, causing economically rational producers to reduce milk production during this period.^[[1]](#footnote-1)^ However, given that the Orthodox fasting periods are scattered throughout the year, such a strategy may at best only partially tackle the mismatch between supply and demand.

Fig B1. Rainfall and agricultural seasons and the timing of main Orthodox fasts and feasts through the year

|  | Jan. | | | | | Feb. | | March | April | May | | | June | | | July | | Aug. | | | Sept. | | Oct. | Nov. | | Dec. |
| --- | --- | --- | --- | --- | --- | --- | --- | --- | --- | --- | --- | --- | --- | --- | --- | --- | --- | --- | --- | --- | --- | --- | --- | --- | --- | --- |
| Rainfall pattern |  | | | | | **Dry period** | | | | | | | **Summer rains** | | | | | | | | | |  | | | |
| Agricultural seasons |  | | | | | | | | | | | | **Lean season** | | | | |  | | | | | **Main harvest season** | | | |
| Orthodox fasts and feasts |  | 1 | 2 |  | 3 | | 4 | | | | 5 | 6 | | 7 | 8 | | 9 | | 10 | 11 | | 12 | | | 13 | |

*Note*. (1) Christmas, (2) Epiphany, (3) Nineveh fast, (4) Lent fast, (5) Easter, (6) Ascension, (7) Pentecost, (8) Apostles fast, (9) Feast of the Apostles, (10) Felseta fast, (11) Assumption feast, (12) Ethiopian New Year, (13) Advent fast.

The dates for Lent, Easter, and associated fasts and feasts are not fixed calendar dates, but can vary by several weeks from year-to-year based on the lunar calendar.

Fig B2. Variation in average daily milk production per cow in milk through the year, liters


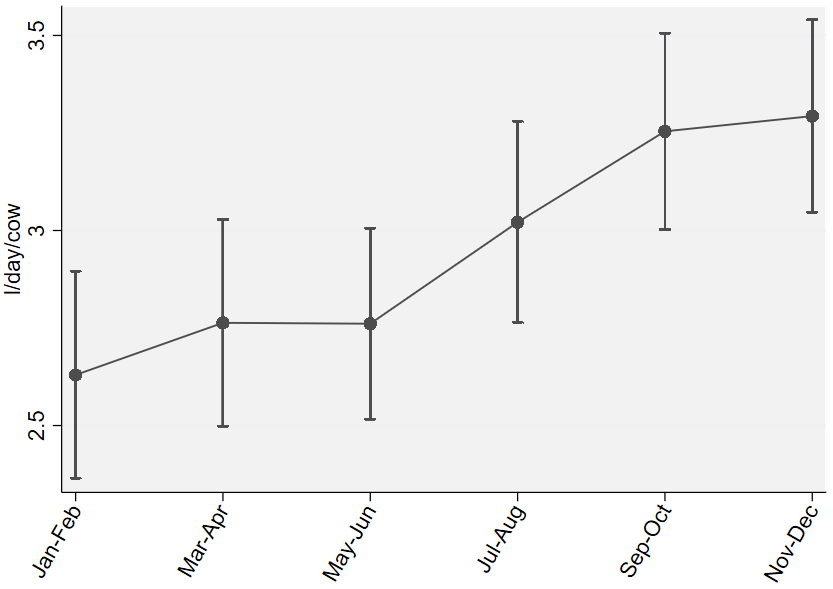


*Note*. Whisker-plots around points are 95-percent confidence intervals.

Fig B3. Variation in local and cross-bred cows in milk through the year, share

| ****Local**** | ****Cross-bred**** |
| --- | --- |
| 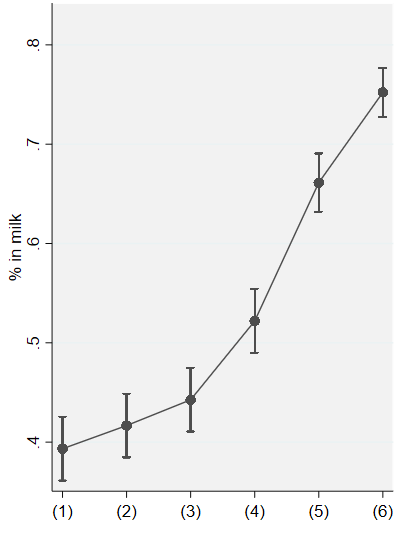 | 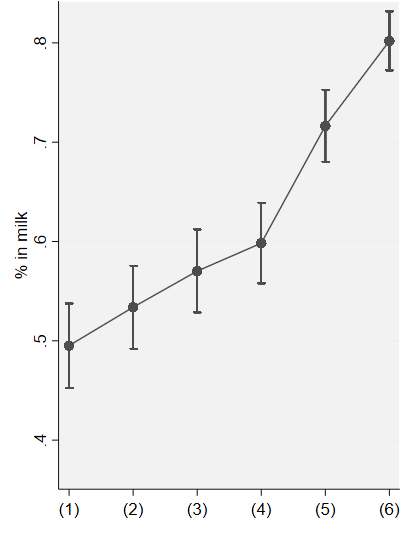 |
| (1) Jan-Feb; (2) Mar-Apr; (3) May-Jun; (4) Jul-Aug; (5) Sep-Oct; (6) Nov-Dec. | |

*Note*. Whisker-plots around points are 95-percent confidence intervals.

1. Gizaw S, Ebro A, Tesfaye Y, Mekuriaw Z, Mekasha Y, Hoekstra D, et al. Feed resources in the highlands of Ethiopia: A value chain assessment and intervention options. Nairobi: International Livestock Research Institute (ILRI); 2017.

2. Dirks R. Social responses during severe food shortages and famine Current Anthropology. 1980;21(1):21-44.

1. Alternatively, one could argue that it is not producers who adjust to the fasting periods, but that Orthodox fasting practices may have emerged in famine-prone Ethiopia to help reduce the pressure on food supply, as suggested by Dirks [2]. [↑](#footnote-ref-1)
