# Supplementary material for: Fasting, food and farming: Value chains and food taboos in Ethiopia
Source: PLoS One. 2021 Dec 9;16(12):e0259982. doi: 10.1371/journal.pone.0259982 (PMC8659323; doi:10.1371/journal.pone.0259982)
Supplement: S3 Appendix — (DOCX) [file pone.0259982.s003.docx]

As a robustness check, we run two double-hurdle models to assess the impact of fasting on dairy market participation and dairy sales volumes. The results are presented in Table C1. As expected, fasting only seems to significantly impact the decision to sell milk and not the subsequent volume of milk sold.

Table C1. Effect of presence of a long fasting season in the previous month on likelihood to sell dairy products (first hurdle) and total dairy product sales over the month prior to interview, conditional on dairy market participation (second hurdle)

| . | **Milk** | | **Processed milk** | |
| --- | --- | --- | --- | --- |
|  | Likelihood  to sell | Sales volume  (l) | Likelihood  to sell | Sales volume  (kg) |
| Fasting past month (0/1) | -0.85***  (0.26) | 1.63  (1.64) | 39.13  (57.11) | 4.31  (8.26) |
| Household controls | Yes | | Yes | |
| Observations | 855  -1750.57 | | 855  -1982.24 | |
| Log likelihood |  |  |  |  |

*Note*. For a complete list of controls, see Supporting information S1. Total dairy production is not included as a control variable. Cluster robust standard errors are in parentheses. *** p<0.01, ** p<0.05, * p<0.1
