# Supplementary material for: Fasting, food and farming: Value chains and food taboos in Ethiopia
Source: PLoS One. 2021 Dec 9;16(12):e0259982. doi: 10.1371/journal.pone.0259982 (PMC8659323; doi:10.1371/journal.pone.0259982)
Supplement: S4 Appendix — (DOCX) [file pone.0259982.s004.docx]

**Fig D1. Impact of a fasting day on likelihood of daily consumption of different food groups for household head and spouse**


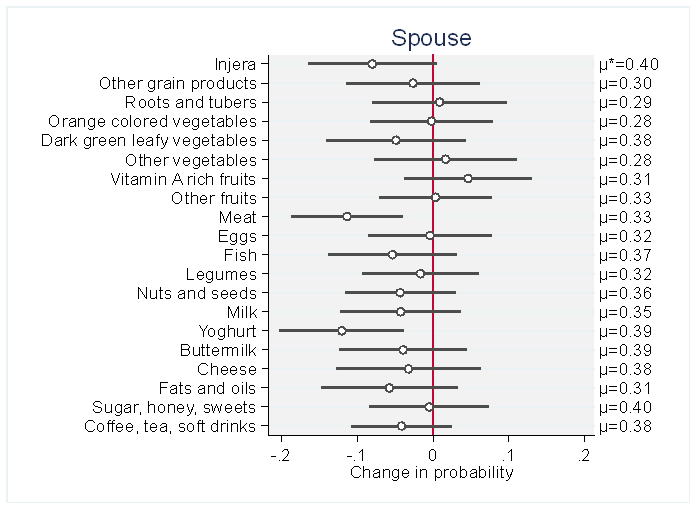
*
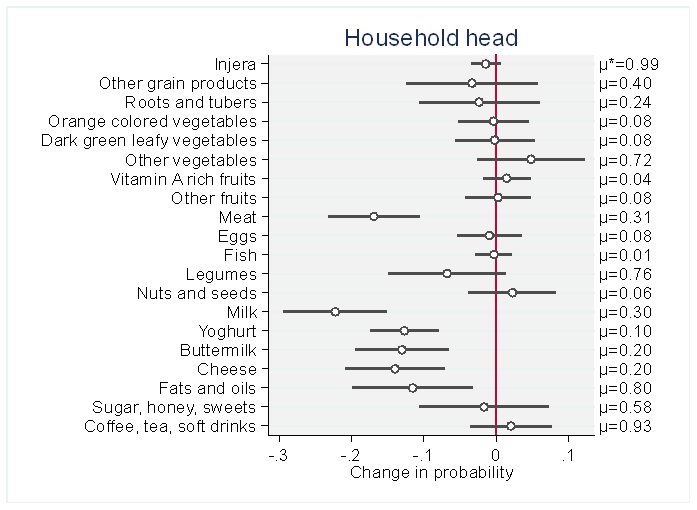
* **Household head Spouse**

*Note*. *µ denotes the non-fasting mean of the dependent variable. Coefficients of the fasting day indicator are shown along with cluster robust 95-percent confidence intervals. For complete list of controls, see Supporting information S1.
