# Supplementary material for: Fasting, food and farming: Value chains and food taboos in Ethiopia
Source: PLoS One. 2021 Dec 9;16(12):e0259982. doi: 10.1371/journal.pone.0259982 (PMC8659323; doi:10.1371/journal.pone.0259982)
Supplement: Attachment — Submitted filename: Response to Reviewer.docx [file pone.0259982.s005.docx]

Dear Reviewer, Dear Prof. Dr. Alafia Samuels

We would like to thank you for your valuable comments on our manuscript. The constructive feedback has been very helpful in guiding our revision. We think the modifications have improved the paper.

In this reply letter, we provide a detailed account of the changes we have made and we explain how we have addressed each comment. Next to this letter, we also provide you with the revised manuscript. We have indicated the changes using "Track Changes”.

We hope you are satisfied with the way we have addressed these comments. We would be glad to answer any further queries, and adjust the paper if more questions or suggestions would arise.

Kind regards,

the authors

**Comment 1**

Page 3, Line 46
Needs to be qualified. Likely true in low income countries, but in upper middle income and high income countries with high meat consumption. “There is a clear link between high intake of red and processed meats and a higher risk for heart disease, cancer, diabetes and premature death” Hu, Harvard

**Reply:** We agree that this statement has to be nuanced and addressed it accordingly in the manuscript:

*“This reduction is unfortunate, as there is a strong link between the consumption of (a reasonable amount of) ASF and improved nutritional outcomes in low income countries^1^(Hoddinott, Headey, & Dereje, 2015).”*

*By contrast, long-term consumption of increasing amounts of red and processed meats has been linked to a higher risk for heart disease, cancer, diabetes and premature death in upper middle and high income countries (Battaglia Richi et al., 2015; Qian et al., 2020; Ranabhat et al., 2020).*

**Comment 2**

Page 4 line 82

Describe the fasting, is it like Ramadan where you eat at night? What, how much and when are they allowed to eat during the fasting.

**Reply:** We included the necessary descriptions of the Orthodox fasting rules on page 3

*“During several fasting periods annually, Orthodox members are to refrain from consuming ASF and therefore adhere to vegan dietary patterns throughout the entire day. Additionally, observants are required to refrain from eating or drinking before noon or the Liturgy and take no more than one meal a day during these fasting periods (Boylston, 2013; Knutsson & Selinus, 1970).*

**Comment 3**

Page 5, line 105-107
Are we using ASF and dairy interchangeably. ASF can include poultry which is not dairy. Perhaps an explanation of what is ASF in Ethiopia. What is the normal diet – we have no idea so far

**Reply:** We agree that this is not clearly described yet in the paper. Therefore we added the following paragraph in the introduction:

*“We study the effects of fasting practices, focusing on the case of dairy production and consumption in Ethiopia, the second most populous country in Africa. Ethiopian diets are mainly composed of cereals (especially teff), roots, tubers and pulses. The consumption of fruit and vegetables as well as ASF are low and cereals remain the major contributors to dietary energy and protein (Sheehy, Carey, Sharma, & Biadgilign, 2019). Annual consumption of ASF per adult equivalent vary around 20 kg, of which dairy products represent 71%, beef 16% and mutton and goat 6%. Chicken, honey, eggs and fish products are less frequently consumed whereas pork is rarely consumed because of religious customs (Abegaz, Hassen, & Minten, 2018).”*

**Comment 4**

Page 7 line paragraph 1

What is the dietary pattern and calories consumed during fasting vs non-fasting for the adults and for the children

**Reply:** Only 2 of the 5 papers mentioned in this section detail dietary intake - consumption (yes/no) of different food groups 24h before the survey - in and outside Lent fasting for children aged 6-23 months (i.e. Desalegn, Lambert, Riedel, Negese, and Biesalski (2019)) and lactating mothers (i.e. Desalegn, Lambert, Riedel, Negese, and Biesalski (2018). None of the 5 papers detail calories consumed.

The paper by Desalegn et al. (2019) conducted a longitudinal community-based survey during and outside the Lent fasting period in the rural Genta Afeshum woreda. 24-h recall data points were collected to assess, the minimum acceptable diet (MAD), the minimum dietary diversity (MDD) and the minimum meal frequency (MMF) for 6-23-month-old children for both fasting and non-fasting lactating mothers. The authors found no significant difference in the consumption of any of the seven food groups (i.e. grains, roots and tubers; pulses and nuts; other fruits and vegetables; dairy products; flesh foods; eggs; vitamin A-rich fruits and vegetables) for children of fasting mothers during and outside the Lent fasting period. A different story emerges for children of non-fasting mothers. For those children, the authors report a significantly higher proportion of children consuming grains, roots and tubers, pulses and nuts, other fruits and vegetables and eggs outside the Lent fasting period resulting in a significantly higher proportion of children of non-fasting mothers reaching the MAD and MDD criteria outside Lent than during Lent. Furthermore, the authors found no significant difference in the consumption of any of the seven food groups (i.e. grains, roots and tubers; pulses and nuts; other fruits and vegetables; dairy products; flesh foods; eggs; vitamin A-rich fruits and vegetables) during the Lent fasting period between children born of fasting and non-fasting mothers as well as the proportion of children of fasting and non-fasting mothers’ sub-groups who fulfilled the MMF, MDD, and MAD standards during the Lent fasting period. Furthermore, the proportion of children who fulfilled the MDD and MAD significantly increased in the non-fasting mothers sub-group compared to the fasting mothers sub-group during the non-fasting period (see the print screen of Table 4 below originating from the article).

.

The paper of Desalegn et al. (2018) assessed the dietary pattern of lactating mothers with children aged between 6 to 23 months old in the Genta Afeshum woreda both during and outside Lent fasting. They found that the proportion of lactating (fasting and non-fasting) mothers who consumed ASFs was significantly lower during Lent than outside Lent. For fasting lactating mothers, they did not observe a significant difference in the proportion of mothers who consumed pulses, dark green leafy vegetables, vitamin A-rich fruits and vegetables and other vegetables groups between Lent and outside Lent. For non-fasting mothers the consumption of pulses and other vegetables groups was also not significantly different between Lent and non-Lent. However, a lower proportion of non-fasting mothers was consuming dark green leafy vegetables outside Lent as compared to during Lent fasting (see the print screen of Table 5 below originating from the article).

Kumera, Tsedal, and Ayana (2018) assessed dietary diversity cross-sectionally for children aged 6–23 months of Orthodox Christian mothers/caregivers in the Dejen district during the Lent fasting period. They found that 98.1% of the mothers/caregivers indicated to have fed their children grains, roots and tubers, 98% fed eggs, 87.9% fed legumes and nuts, 78.3% fed other fruits and vegetables, 17.5% fed vitamin A-rich fruits and vegetables and 7.5% fed dairy products. The authors also found that the mean Dietary Diversity Score (DDS) during fasting was 3.13 (± 0.87) and that 86.4% of the children had a low DDS (< 4 food groups). No comparison was made for the children’s diets during non-fasting periods.

In the paper by Kim et al. (2019) Orthodox Christian households with children 6–23 months were surveyed only during the Lent fasting period in 20 woredas (districts) belonging to three western zones of Amhara region (Awi, North Gondor, and West Gojjam). They found that 87.8% of the children consumed grains, roots and tubers, 55.9% consumed legumes and nuts, 17.6% consumed dairy, 9.7% consumed vitamin A-rich fruits and vegetables, 8% consumed eggs and 2.1% consumed flesh food and 1.5% consumed other fruits and vegetables.

Finally in the study of Potts, Mulugeta, and Bazzano (2019), the consumption (yes/no) of different ASFs (eggs, fish, yoghurt, cheese, milk, meat and organ meats) by 6 to 36-month-old children as well as the MDD was assessed cross-sectionally in four regions of Ethiopia (Afar, Amhara, Benishangul-Gumuz, and Tigray) without detailing differences in consumption patterns for fasting or non-fasting periods.

Given the interesting consumption difference within and outside fasting periods, we added more background for the studies conducted by Desalegn et al. (2019) for children and Desalegn et al. (2018) for lactating mothers in the manuscript.

*“Other studies have examined the specific impact of fasting on the milk intake of young children (Desalegn et al., 2019; Kim et al., 2019; Kumera et al., 2018) and on lactating mothers (Desalegn et al., 2018) in Ethiopia. While pregnant and lactating women and children below the age of seven years in principle are exempt from fasting (Knutsson & Selinus, 1970), all of these studies found negative repercussions of Orthodox fasting practices on dietary diversity and intake of ASF. As such, Desalegn et al. (2019) who compared dietary intake in and outside Lent fasting for children aged 6-23 months old, found that while there was no significant difference in the consumption of 7 food groups (i.e. grains, roots and tubers; pulses and nuts; other fruits and vegetables; dairy products; flesh foods; eggs; vitamin A-rich fruits and vegetables) for children of fasting mothers during and outside the Lent fasting period, a significantly higher proportion of children from non-fasting mothers was consuming grains, roots and tubers, pulses and nuts, other fruits and vegetables and eggs outside the Lent fasting period resulting in a significantly higher proportion of children of non-fasting mothers reaching the minimum acceptable diet and minimum dietary diversity criteria outside Lent than during Lent. This study thus shows that maternal fasting adherence influences children’s dietary intake and nutritional status significantly, even outside of official fasting periods. Furthermore, Desalegn et al. (2018) observed when comparing diets of fasting and non-fasting lactating mothers, that the proportion of lactating (fasting and non-fasting) mothers who consumed ASF was significantly lower during Lent than outside Lent, while a lower proportion of non-fasting mothers was consuming dark green leafy vegetables outside Lent as compared to during Lent fasting.* *No significant difference was found in the proportion of fasting lactating mothers who consumed pulses, dark green leafy vegetables, vitamin A-rich fruits and vegetables and other vegetables groups between Lent and outside Lent and the proportion of non-fasting mothers consuming pulses and other vegetables groups. Kim et al. (2019), who studied ASF consumption by children aged 6 to 23 months during Lent, observed that only a quarter of surveyed children consume ASF during the Lent fasting period, even though 80 percent of the surveyed households reported owning livestock. They found that caregivers are reluctant to feed ASF to their children during Lent, even when livestock products are available, because they fear the disapproval of their neighbors or the contamination of cooking utensils with non-fasting foods. A recent study by Potts et al. (2019) even found that Muslim children had a higher probability (20%) of having consumed ASF as compared to children from Orthodox households and households adhering to other religions.”*

**Comment 5**

Page 9 line 222
Is there any IRB in Ethiopia? Perhaps at the university? Was any consent sought from any government entity, like the Ministry of Health or Agriculture?

**Reply:** There are IRBs in Ethiopia and we sought approval from the IRB committee of the International Food Policy Research Institute based in Addis Ababa**.** The survey was done in collaboration with the Ethiopian Development Research Institute, a think tank linked to the government of Ethiopia. We emphasized this now more clearly in the manuscript:

*“The Institutional Review Board of the International Food Policy Research Institute granted approval for this study. The survey was carried out in collaboration with the Ethiopian Development Research Institute, a think tank linked to the government of Ethiopia. Consent was informed and written.”*

**Comment 6**

Line 331-337

Could this be a cohort effect where the pattern is changing, and younger persons who are more exposed, are less adherent. This might also explain the difference in these estimates vs previous studies.

**Reply:** It is indeed possible that fasting practices might have changed over time. We include this potential cohort effect now in the manuscript.

*“Table 5 summarizes the extent of participation in fasting periods throughout the year for Orthodox households. The number of fasting days on a yearly basis varies from 5.2 days for young children to 142 days for elderly people. The fasting adherence of adults is around 140 days and thus 26 to 110 days below the estimates of 166 to 250 fasting days per year reported elsewhere (Abegaz et al., 2018; Bachewe, Minten, & Yimer, 2017; Francesconi, Heerink, & D'Haese, 2010).* *Both observations could indicate that fasting practices are changing over time and that younger people - who are increasingly exposed to more liberal and thus less strict fasting practices - become less adherent.”*

**Comment 7**

Line 483

Still no overall depiction of calories and dietary patterns during fasting and non-fasting for adults and children, remote and non-remote

**Reply:** Regarding the effect of fasting on the consumption of individual food groups beyond dairy, we focus exclusively on the youngest child in the household as the full analysis of dietary patterns in the household is considered beyond the scope of the paper. The paper looks at the combination of production and consumption decisions in dairy-producing households, so the consumption of non-dairy food groups does not add much information in that respect. Additionally, as consumption patterns were but a small part of the study, the type of non-dairy consumption data collected is not detailed enough to provide a full mapping of dietary patterns at the household level. Such consumption data was not collected for all members of the household, only for the youngest child, the household head and the spouse. On top of that, the questionnaire only contained yes or no question with respect to consumption of different food groups, which means that it is unfortunately not possible calculate the amount of calories consumed on a daily basis.

The effect of fasting on food groups beyond dairy for the youngest child is shown to assess whether one of the surprising results of the paper (higher consumption of milk during fasting periods) also holds for other ASF and non-ASF food groups. We are able to show that it does not and speculate this could be due to the fact that ASF food groups are prepared less at the household level, but cannot explicitly prove this due to the reasons mentioned above.

In Appendix D, we now also show the same type of graphs for the household head and the spouse, for information (see below). They show that, among adults in the household, fasting days primarily impact the likelihood of consuming animal-sourced foods with higher impacts reported for the household head than for the spouse. They also show that the likelihood of consuming certain food items on a given day, can vary widely between the household head and the spouse.

 **Household head	Spouse**

***Fig D1. Impact of a fasting day on likelihood of daily consumption of different food groups for household head and spouse***

*Note. *µ denotes the non-fasting mean of the dependent variable. Coefficients of the fasting day indicator are shown along with cluster robust 95-percent confidence intervals. For complete list of controls, see Supporting information S1.*

Abegaz, G. A., Hassen, I. W., & Minten, B. (2018). *Consumption of animal-source foods in Ethiopia: Patterns, changes, and determinants* (ESSP Working paper No. 113). Retrieved from International Food Policy Research Institute (IFPRI) and Ethiopian Development Research Institute (EDRI) Published: <http://ebrary.ifpri.org/utils/getfile/collection/p15738coll2/id/132252/filename/132463.pdf>

Bachewe, F., Minten, B., & Yimer, F. (2017). *The rising costs of animal-source foods in Ethiopia: Evidence and implications* (ESSP Working paper No. 108). Retrieved from International Food Policy Research Institute (IFPRI) and Ethiopian Development Research Institute (EDRI) Published: <http://ebrary.ifpri.org/utils/getfile/collection/p15738coll2/id/131369/filename/131580.pdf>

Battaglia Richi, E., Baumer, B., Conrad, B., Darioli, R., Schmid, A., & Keller, U. (2015). Health Risks Associated with Meat Consumption: A Review of Epidemiological Studies. *International journal for vitamin and nutrition research. Internationale Zeitschrift fur Vitamin- und Ernahrungsforschung. Journal international de vitaminologie et de nutrition, 85*(1-2), 70-78. doi:10.1024/0300-9831/a000224

Boylston, T. (2013). Food, life, and material religion in Ethiopian Orthodox Christianity. In J. Boddy & M. Lambek (Eds.), *A companion to the anthropology of religion* (pp. 257–274). Chichester: John Wiley and Sons.

Desalegn, B. B., Lambert, C., Riedel, S., Negese, T., & Biesalski, H. K. (2018). Ethiopian Orthodox fasting and lactating mothers: Longitudinal study on dietary pattern and nutritional status in rural Tigray, Ethiopia. *Int J Environ Res Public Health, 15*(8), 1767. doi:10.3390/ijerph15081767

Desalegn, B. B., Lambert, C., Riedel, S., Negese, T., & Biesalski, H. K. (2019). Feeding practices and undernutrition in 6-23 month-old children of Orthodox Christian mothers in rural Tigray, Ethiopia: Longitudinal study. *NUTRIENTS, 11*(1), 138. doi:10.3390/nu11010138

Francesconi, G. N., Heerink, N., & D'Haese, M. (2010). Evolution and challenges of dairy supply chains: Evidence from supermarkets, industries and consumers in Ethiopia. *Food Policy, 35*(1), 60-68. doi:10.1016/j.foodpol.2009.06.003

Hoddinott, J., Headey, D., & Dereje, M. (2015). Cows, missing milk markets and nutrition in rural Ethiopia. *Journal of Development Studies, 51*(8), 958-975. doi:10.1080/00220388.2015.1018903

Kim, S. S., Nguyen, P. H., Tran, L. M., Abebe, Y., Asrat, Y., Tharaney, M., & Menon, P. (2019). Maternal behavioural determinants and livestock ownership are associated with animal source food consumption among young children during fasting in rural Ethiopia. *Maternal and Child Nutrition, 15*(2), e12695. doi:10.1111/mcn.12695

Knutsson, K. E., & Selinus, R. (1970). Fasting in Ethiopia. An anthropologican and nutritional study. *The American Journal of Clinical Nutrition, 23*(7), 956-969. doi:10.1093/ajcn/23.7.956

Kumera, G., Tsedal, E., & Ayana, M. (2018). Dietary diversity and associated factors among children of Orthodox Christian mothers/caregivers during the fasting season in Dejen District, North West Ethiopia. *Nutrition and Metabolism, 15*, 16. doi:10.1186/s12986-018-0248-0

Potts, K. S., Mulugeta, A., & Bazzano, A. N. (2019). Animal source food consumption in young children from four regions of Ethiopia: Association with religion, livelihood, and participation in the Productive Safety Net Program. *NUTRIENTS, 11*(2), 354. doi:10.3390/nu11020354

Qian, F., Riddle, M. C., Wylie-Rosett, J., & Hu, F. B. (2020). Red and Processed Meats and Health Risks: How Strong Is the Evidence? *DIABETES CARE, 43*(2), 265-271. doi:10.2337/dci19-0063

Ranabhat, C. L., Park, M.-B., & Kim, C.-B. (2020). Influence of Alcohol and Red Meat Consumption on Life Expectancy: Results of 164 Countries from 1992 to 2013. *NUTRIENTS, 12*(2), 459.

Sheehy, T., Carey, E., Sharma, S., & Biadgilign, S. (2019). Trends in energy and nutrient supply in Ethiopia: a perspective from FAO food balance sheets. *Nutrition Journal, 18*(1), 46. doi:10.1186/s12937-019-0471-1
